# Understanding the Tissue Specificity of ZIKV Infection in Various Animal Models for Vaccine Development

**DOI:** 10.3390/vaccines10091517

**Published:** 2022-09-13

**Authors:** Suyeon Kim, Ha Youn Shin

**Affiliations:** Department of Biomedical Science and Engineering, Konkuk University, Seoul 05029, Korea

**Keywords:** ZIKV, vaccine, tissue tropism, animal model, mouse, non-human primate

## Abstract

Zika virus (ZIKV) is an arthropod-borne virus that belongs to the Flavivirus genus and is principally transmitted by *Aedes aegypti* mosquitoes. ZIKV infection often causes no or only mild symptoms, but it can also trigger severe consequences, including microcephaly in infants and Guillain-Barré syndrome, uveitis, and neurologic manifestations in adults. There is no ZIKV vaccine or treatment currently approved for clinical use. The primary target of ZIKV infection has been recognized as the maternal placenta, with vertical transmission to the fetal brain. However, ZIKV can also spread to multiple tissues in adults, including the sexual organs, eyes, lymph nodes, and brain. Since numerous studies have indicated that there are slightly different tissue-specific pathologies in each animal model of ZIKV, the distinct ZIKV tropism of a given animal model must be understood to enable effective vaccine development. Here, we comprehensively discussed the tissue specificity of ZIKV reported in each animal model depending on the genetic background and route of administration. This review should facilitate the selection of appropriate animal models when studying the fundamental pathogenesis of ZIKV infection, thereby supporting the design of optimal preclinical and clinical studies for the development of vaccines and therapeutics.

## 1. Introduction

### 1.1. Epidemiology and Clinical Manifestation of ZIKV Outbreaks

**Figure 1:**
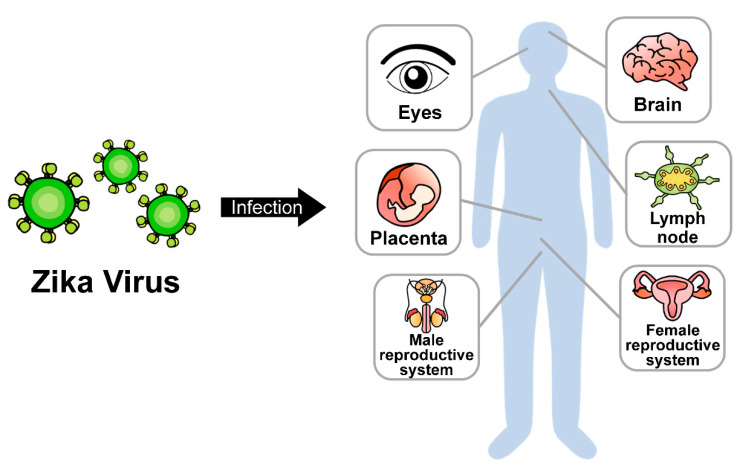
Dissemination of ZIKV in multiple organs.

Zika virus (ZIKV) is a mosquito-transmitted RNA virus; it belongs to genus *Flavivirus* in the *Flaviviridae* family and is closely related to the dengue, yellow fever, Japanese encephalitis, and West Nile viruses [1]. Like other flaviviruses, ZIKV is enveloped and icosahedral and has an 11-kb positive single-stranded RNA genome. ZIKV was first isolated in 1947 from a rhesus monkey from the Zika forest of Uganda [2]. Sporadic ZIKV epidemic outbreaks were reported in French Polynesia (2013–2014), Brazil (2015), and South and Central America (2016) [3]. After human-to-human transmission was reported in 2016, the World Health Organization (WHO) declared ZIKV to be a major threat to public health. So far, two major strains of ZIKV have been identified: the African and Asian strains. Both are transmitted primarily via mosquitoes and can also be transmitted through mother-to-fetus transmission, breastfeeding, sexual contact, or blood transfusion [4]. The primary target tissues of ZIKV infection have been recognized as the maternal placenta and fetal brain. However, ZIKV can also be transmitted to other adult tissues, including the reproductive system, brain, eyes, and lymph nodes (Figure 1). ZIKV infection causes several clinical complications, including congenital microcephaly, autoimmune Guillain-Barré syndrome, and ocular diseases, such as uveitis and unilateral acute maculopathy [5,6,7,8].

### 1.2. Current Status of ZIKV Vaccine Development

Currently, there is no licensed ZIKV vaccine on the market. Since the primary target for ZIKV vaccination is pregnant women, a developed vaccine must be safe and effective for both pregnant women and their fetuses. As of July 2022, several ZIKV vaccines are undergoing phase 1 and 2 clinical trials [9,10,11,12] (Table 1). The major types of candidate vaccines include DNA, mRNA, inactivated virion, and peptide vaccines. The most commonly used immunogen is prM/E, which is the envelope protein of ZIKV. In early 2017, the first phase 2 clinical trial was approved for the ZIKV vaccine candidate VRC5283, which was a DNA-based vaccine developed by the National Institute of Allergy and Infectious disease (NIAID). VRC5283 consists of a DNA plasmid encoding the E and PrM proteins of the Asian strain of ZIKV [9,10]. Preclinical studies demonstrated that it could elicit neutralizing antibodies against ZIKV in both murine and non-human primate models.

Although various ZIKV vaccine candidates have been developed in the past few years, many challenges have limited their progression to clinical trials. The primary recipients of ZIKV vaccination would be pregnant women, but it has proven difficult to recruit participants for clinical trials. Safety and ethical issues mean that it can take a long time to recruit sufficient healthy study volunteers in ZIKV epidemic regions. Researchers have also found it difficult to conduct preclinical studies, given that ZIKV susceptibility and pathological features exhibit slight differences between humans and animal models of various genetic backgrounds. It is very important that we understand the pathological features of each animal model for ZIKV infection, as this will enable optimal animal models to be selected for preclinical studies and support successful progression to clinical studies.

### 1.3. Animal Models for Studying ZIKV

Several animal models have been used to explore the infection, transmission, etiology, and immune mechanisms of ZIKV. The most commonly used animal models for ZIKV studies are mice and nonhuman primates.

#### 1.3.1. Mouse Models

Mice are the most widely used laboratory animals due to their low cost of breeding, short generation time, numerous litters, and small size. However, wild-type or immunocompetent mice, such as C57BL/6, ICR (CD-1), and SJL mice, are naturally resistant to ZIKV infection [3]. In humans, ZIKV infection stimulates the interferon (IFN)-α/β receptors and activates the Janus kinase/signal transducer and activator of transcription (JAK/STAT) pathway to induce IFN-stimulated genes (ISGs) that are essential for innate immunity [13]. However, the NS5 protein of ZIKV binds and degrades STAT2 to disrupt IFN signaling [14]. In contrast, ZIKV NS5 does not bind efficiently to murine STAT2; this prevents the virus from escaping the innate immune system and allows mice to resist ZIKV infection.

To address this issue, researchers have often administered anti-IFN-α/β receptor antibodies to block IFN signaling in immunocompetent mice to improve ZIKV susceptibility [15]. Gordon and colleagues also recently created human STAT2 knock-in (KI) mice by introducing human *STAT2* into the mouse *Stat2* locus [16]. Alternatively, many research groups have used immunocompromised mice with genetic deficiencies in the IFN pathway, including IFN-α/β receptor knockout (KO) (A129), IFN-α/β/γ receptor KO (AG129), Stat2 KO, Irf3/Irf5 double KO, and Irf3/Irf5/Ifr7 triple KO mice [17,18,19,20,21,22,23,24]. These immunodeficient mice are susceptible to ZIKV infection and display various viral pathologies found in humans [17,19,25,26]. Indeed, these mouse models have been used for the preclinical evaluation of vaccine candidates. Ifnar1^−/−^ mice were employed to test the protective efficacy of a DNA vaccine candidate encoding ZIKV prM/E (GLS-5700) against testicular damage caused by ZIKV infection [27]. Both A129 mice and anti-Ifnar1 antibody-treated immunocompetent mice were used to evaluate the protective efficacy of a prM/E mRNA vaccine and a live-attenuated vaccine candidate against placental and fetal damage [28,29]. CD-1 and AG129 mice were used to evaluate the preclinical immunogenicity and efficacy of a purified inactivated ZIKV vaccine candidate (PIZV) [30]. Nevertheless, the fetal developmental process and placental structure in mice differ from those in humans [31,32,33,34]. Moreover, these mouse models cannot represent the developmental processes of the human brain and central nervous system [34,35] and therefore do not display ZIKV-related Guillain-Barré syndrome. These distinct features might limit the usefulness of mouse models in this context.

#### 1.3.2. Nonhuman Primate Models

Nonhuman primates are very similar to humans in terms of their immune system, reproduction, and developmental processes. Several reports have shown that nonhuman primates of different species, including rhesus, cynomolgus, and pigtailed macaques, can be infected with various ZIKV strains either subcutaneously or intravenously (Table 2). A preclinical study was also conducted in rhesus macaques to demonstrate the efficacy of the DNA vaccine candidate VRC5283 [36]. The persistent efficacy of a ZPIV vaccine candidate was evaluated in rhesus monkeys [37]. Although nonhuman primates offer many advantages arising from their close resemblance to humans, their use in preclinical studies is limited by ethical issues, high breeding costs, and limitations in group size. Nevertheless, it is evident that the use of nonhuman primate models can enable accurate research for ZIKV [17].

Clearly, each animal model has advantages and limitations for studying ZIKV. In this review, we comprehensively discuss the multiple tissue tropism of ZIKV and the tissue-specific symptoms revealed in each animal model. This review will provide insight into the selection of animal models and tissue types suitable for preclinical studies seeking to develop vaccines and treatments for ZIKV outbreaks.

## 2. ZIKV Transmission from Maternal Placenta to Fetal Brain

A key feature of congenital ZIKV syndrome is microcephaly, wherein an infant displays a smaller-than-normal head due to immature brain development during pregnancy. Severe microcephaly is correlated with mental retardation, learning disability, and poor motor function [38]. ZIKV infection in pregnant women has received considerable attention due to the vertical transmission of ZIKV from the maternal placenta to fetuses, resulting in high rates of microcephaly [39,40]. Several studies have demonstrated that ZIKV can replicate in placental cells [41,42,43] and in fetal endothelial cells upon the infection of the placental barrier [44]. Accordingly, many researchers have focused primarily on understanding the mechanisms of ZIKV infection in the placenta and fetus when seeking to develop vaccines and therapeutics.

### 2.1. Mice

Mouse models have shown slight differences in viral susceptibility to and clinical manifestations of ZIKV infection in the maternal placenta and fetus depending on the genetic background and/or route of administration. For example, the subcutaneous injection of ZIKV in immunocompetent mice, such as C57BL/6, CD-1, or SJL mice, yielded insignificant clinical signs and no viral replication due to the intact innate immunity [21,26,45]. However, several studies found that intravenous or intrauterine ZIKV infection in these mice induced viral replication in the placenta and the resorption of fetuses [26] (Table 2). When C57BL/6 mice were inoculated with a high dose of ZIKV via an intravenous route at embryonic day 9.5 (E9.5), fetuses were resorbed at day E17.5 even though the ZIKV RNA was only sparsely detected in the fetal head [46]. When ZIKV was inoculated directly into the uterus of pregnant CD-1 mice, viral RNA was observed in the uterine horn, placenta, and fetal head [47]. This inoculation also increased fetal abortion, placental inflammation, and IFN-β levels. In ICR mice, the injection of ZIKV into the embryonic brains at E15.5 led to fetal death and microcephaly [24,48]. The intravenous injection of ZIKV into SJL mice also induced the congenital malformation of the brain [49]. Human STAT2 KI mice and immunodeficient mice showed more enhanced viral susceptibility and evident clinical symptoms. Even the subcutaneous injection of ZIKV into human STAT2 KI mice resulted in higher levels of ZIKV RNA in the placenta and fetal heads compared with the immunocompetent wild-type mice [16]. The subcutaneous injection of ZIKV into IFN-deficient A129 and AG129 mice resulted in a small fetal head and the detection of viral RNA in the placenta and fetal head [50,51]. Taken together, these results indicate that mice are naturally resistant to ZIKV infection but that mouse models may exhibit human-like symptoms such as microcephaly depending on their genetic background and/or the route of ZIKV administration.

### 2.2. Non-Human Primates

Compared with the mouse models, the pathological symptoms of ZIKV infection during pregnancy in nonhuman primates are more analogous to those of humans [52]. Several studies have shown that the subcutaneous injection of ZIKV in rhesus monkeys induced robust placental and fetal inflammation, placental dysfunction, and fetal neuropathology, such as neural progenitor cell dysfunction and neuronal apoptosis [53,54]. The direct inoculation of ZIKV into the amniotic fluid at early pregnancy resulted in more severe symptoms, including loss of neural precursor cells and early fetal death [55]. The subcutaneous inoculation of ZIKV into pregnant pigtail monkeys induced fetal brain damage, such as fetal brain growth arrest and a significant loss of fetal neural progenitor cells [56,57]. The intramuscular injection of ZIKV into a rat-sized marmoset induced human-like fetal neurodevelopmental abnormalities and spontaneous fetal loss accompanied by extensive viral replication in the placenta [58]. Although the gestational duration is shorter in these nonhuman primates than in humans, the above-described results indicate that nonhuman primates may recapitulate the human situation for clinical symptoms of ZIKV infection during pregnancy.

**Table 2:** Pathological features of ZIKV infection in maternal placentas and fetuses of animal models.

Animal Species	ZIKV Strain	Route of Administration	Pathologic Features	Reference
**Mouse**				
C57BL/6	PRVABC59	Intravenous	Fetal resorption, little or no viral RNA in placenta and fetal heads	[46]
CD-1	IBH 30656, PRVABC59, FS13025	Intrauterine	Placental dysfunction and inflammation, increased aborted fetus numbers, reduced neonatal brain and cortical thickness	[47]
SJL	ZIKV-BR	Intravenous	Malformations of congenital brain, intrauterine growth restriction, upregulation of apoptosis-related genes	[49]
ICR	GZ01	Intra-ventricular	Fetal death, microcephaly	[48]
Human STAT2 KI	ZIKV-Dak-MA	Subcutaneous	Viral RNA in placenta and fetal heads	[16]
Ifnar1^−/−^	FSS13025	Subcutaneous	Fetal resorption, reduced fetal weight and size	[59]
A129	PRVABC59	Subcutaneous	Viral RNA in placenta, maternal brain, and fetal heads, reduced fetal weight	[51]
AG129	P6-740	Subcutaneous	Viral RNA in placenta and fetal heads, decreased weight and head length	[50]
**Monkey**				
Rhesus macaque	PRVABC59	Subcutaneous	Viral RNA in fetus, increased maternal-placental-fetal inflammatory response, placenta damage, reduced oxygen permeability of the placental villi	[53]
Rhesus macaque	ZIKV-BR	Subcutaneous	Fetal neuropathology, neuroprogenitor apoptosis and gliosis, placental pathology	[54]
Rhesus macaque	SPH 2015	Intra-amniotic	Viral RNA in fetal and placental tissues, early death of fetus, fetal ZIKV neurotropism, neuropathology at the end of gestation	[55]
Pigtail macaque	FSS13025	Subcutaneous	Reduced growth of the fetal biparietal diameter and fetal brain lesions, viral RNA in fetal brain and placenta	[56]
Pigtail macaque	FSS13025, ZIKV-BR	Subcutaneous	Decreased fetal non-cortical brain volume, injury in ependymal epithelium with underlying gliosis, reduced late fetal neuronal progenitor cells in the subventricular zone	[57]
Marmoset	SPH 2015	Intramuscular	Fetal demise and abortion, increase of proinflammatory cytokines, fetal neurocellular disorganization, viral RNA in placenta and fetus	[58]

## 3. ZIKV Infection in Reproductive Organs

### 3.1. Female Animal Models

In addition to vertical transmission from mother to fetus, ZIKV can be transmitted through sexual contact and can cause genital infection. Several research groups have demonstrated that ZIKV can infect the female reproductive organs and detected ZIKV RNA in the female genital tract in animal models, including cervical mucus, vaginal secretions, and ovaries [60,61] (Table 3). Even in immunocompetent C57BL/6 mice, vaginal infection with ZIKV resulted in local viral replication [60]. In immunodeficient Ifnar1^−/−^ and Irf3^−/−^Irf7^−/−^ mice, vaginal ZIKV infection induced higher levels of viral replication. In C57BL/6 mice treated with anti-Ifnar1 monoclonal Ab (mAb) to block the IFN pathway, viral RNA was detected in the vaginal lumen, cervix, ovaries, and uterine horns after intravaginal inoculation of ZIKV [62]. Subcutaneous inoculation of ZIKV in anti-Ifnar1 mAb-treated T-cell-deficient CD8^−/−^ and TCRβδ^−/−^ mice resulted in higher ovarian cell death compared to that in C57BL/6 mice [61]. Transmission of ZIKV from female reproductive organs has also been found in nonhuman primates. In rhesus monkeys, direct injection of ZIKV into the vagina led to preferential viral replication in the female reproductive tract [63]. Even after subcutaneous injection, ZIKV RNA was detected in the vaginal secretions and reproductive tissues of female rhesus and cynomolgus monkeys [64].

### 3.2. Male Animal Models

ZIKV can also infect the male reproductive system. Cohort studies have revealed that the ZIKV RNA can be detected in semen and can persist in the male reproductive tract [65,66,67,68,69]. Persistent ZIKV replication in the male genital tract can cause genitourinary symptoms such as inflammation of the prostate gland, bloody semen, and infertility. The subcutaneous inoculation of ZIKV into C57BL/6 and Ifnar1^−/−^ mice resulted in decreased testis size and testosterone levels and degradation of the seminiferous tubule due to persistent viral replication in semen [15,70] (Table 3). On the other hand, infection of ZIKV via an intraperitoneal route into immunodeficient Ifnar1^−/−^ and AG129 mice caused inflammation in the testis and epididymis, leading to male infertility [23,71,72]. Similarly, ZIKV RNA was detected in monkey testicles after subcutaneous injection [73].

**Table 3:** Effects of ZIKV on reproductive systems in animal models.

Animal Species	ZIKV Strain	Route of Administration	Pathologic Features	Reference
**Female**				
C57BL/6	FSS13025	Intravaginal	Viral RNA in vagina until 4 dpi (local replication)	[60]
Ifnar1^−/−^, Irf3^−/−^Irf7^−/−^	FSS13025	Intravaginal	High viral RNA levels in vagina	[60]
C57BL/6J (+ anti-Ifnar1 mAb)	ZIKV-BR	Intravaginal	ZIKV in the vaginal lumen, cervix, ovaries, uterine horns	[62]
C57BL/6, CD8^−/−^, TCRβδ^−/−^ (+ anti-Ifnar1 mAb)	Dakar 41525, PRVABC59, H/PF/2013	Subcutaneous	Viral replicates in ovary, increased inflammation in the ovary, acute oophoritis	[61]
Rhesus macaque	SPH 2015	Intravaginal	Viral RNA in female reproductive tract, increased cytokine levels in cervicovaginal lavage	[63]
Rhesus macaque, Cynomolgus macaque	Thai, Puerto Rican	Subcutaneous	Viral RNA in female reproductive tissues	[64]
**Male**				
C57BL/6 (+ anti-Ifnar1 mAb)	Dakar 41519, H/PF/2013	Subcutaneous	Viral RNA in the testis and epididymis, decreased testis size, low testosterone level, infected spermatogonia, degradation of seminiferous tubule	[15]
Ifnar^−/−^	MEX2-81	Subcutaneous	Viral RNA and antigen in the epididymal lumen, testicular atrophy, low testosterone level	[70]
Ifnar^−/−^	SMGC-1	Intraperitoneal	Atrophy of the reproductive tract, virus in testicles, increased inflammation in the testis and epididymis	[71]
AG129	PRVABC59	Intraperitoneal	Viral RNA and virus in semen, increased inflammation in testis and epididymis	[72]
AG129	SL1602	Intraperitoneal	Viral RNA in testis and epididymis, increased inflammation in testis	[23]
Cynomolgus macaque	IBH30656, PRVABC59, FSS13025	Subcutaneous	Virus in the testis	[73]

## 4. ZIKV Tropism in Other Tissues

### 4.1. Brain

One unique feature of ZIKV infection is that it preferentially infects the fetal brain, causing congenital or postnatal microcephaly [74]. ZIKV can also affect the adult brain to cause Guillain-Barré syndrome, an autoimmune disease in which the immune system attacks the peripheral nervous system [4,75]. Guillain-Barré syndrome further leads to muscle weakness, paralysis, and ultimately death [76,77,78]. Several animal models can recapitulate the brain or neurological damage seen in humans (Table 4). In C57BL/6 mice treated with anti-Ifnar1 mAb, either the subcutaneous or the intraperitoneal injection of ZIKV induced inflammatory responses and neuronal cell death in the brain and spinal cord [79]. The subcutaneous injection of ZIKV into human STAT2 KI mice resulted in high levels of viral RNA in the brain [16]. However, intraperitoneal infection yielded higher viral susceptibility (100% mortality) than that achieved with subcutaneous infection (40% mortality). In immunodeficient A129 mice, ZIKV RNA was also detected in adult brain tissue after subcutaneous injection, and nuclear fragments were found with necrosis in a portion of the hippocampus [80]. Adult AG129 mice showed high viral susceptibility with severe brain pathology after subcutaneous, intraperitoneal, or foot-pad ZIKV inoculation [18,19,81]. Histopathological examination revealed neutrophil infiltration in the hippocampus, neuronal degeneration, and necrotic cell debris. High viral RNA loads were also observed in the brain and spinal cords. Mice with triple knockout of Irf3, Ifr5, and Irf7 were highly vulnerable to subcutaneous, intraperitoneal, or intravenous ZIKV infection and developed neurological diseases such as hindlimb weakness and paralysis [45]. Taken together, these animal studies clearly demonstrated that ZIKV can damage the adult brain and nervous tissue, although the detailed mechanism underlying Guillain-Barré syndrome remains to be fully elucidated.

### 4.2. Eyes

Several case reports have shown that ZIKV infection can cause ocular diseases, such as conjunctivitis and optic nerve abnormalities [82,83]. These ocular pathologies have also been observed in animal models (Table 4). The subcutaneous injection of ZIKV into C57BL/6 mice preferentially infected the cornea and retina, resulting in inflammation of the optic nerves and the distortion of eye structures [84]. The direct inoculation of ZIKV into the eyes of C57BL/6 and ISG15 knockout mice yielded ocular abnormalities, including pigment clumping and retinal pigment epithelium (RPE) atrophy [85]. ISG15 knockout mice showed more severe retinitis and retinal cell death with higher levels of ZIKV RNA in the retina. ZIKV inoculation to the anterior chamber of the eye or by intraperitoneal route in C57BL/6 and Ifnar1 knockout mice induced innate immune responses and increased intraocular pressure (IOP), which is a hallmark of glaucoma (a major cause of blindness) [86]. Posterior retinal cell death was observed even following ZIKV infection in the anterior part of the eye, suggesting that ZIKV may spread from the anterior to posterior part of the eye [87]. Together, these results indicate that ZIKV can affect the optic nerve, which is part of the central nervous system.

### 4.3. Lymph Nodes

ZIKV infection in lymph nodes was commonly found in nonhuman primates (Table 4). Following the subcutaneous injection of ZIKV into rhesus monkeys, ZIKV could persist up to 72 days in the lymph nodes even after the virus had been cleared from the peripheral blood [88]. Among the various cell types within lymphoid tissues, ZIKV RNA was mainly found in the macrophage and B cell subsets, whereas little or no ZIKV RNA was found in the subsets of dendritic cells and T cells [89]. Intravenous inoculation of ZIKV into rhesus monkeys clearly induced hyperplasia of the lymph nodes, but the activation of lymphocytic cells was limited to the monocyte and B cell subsets [90]. Subcutaneous ZIKV injection in pigtail monkeys also resulted in an innate immune response, with increased proinflammatory cytokine levels and long-term ZIKV persistence in the lymph nodes [91]. Vaginal inoculation of ZIKV-infected semen into baboons yielded the persistence of ZIKV RNA in lymph nodes [92]. This persistent ZIKV infection in lymph nodes and lymphocytic inflammatory responses found in nonhuman primates recapitulate the pathogenic features of ZIKV infection in humans.

**Table 4:** Pathologic features of ZIKV dissemination in brain, eyes, and lymph nodes of animal models.

Animal Species	ZIKV Strain	Route of Administration	Pathologic Features	Reference
**Brain**				
C57BL/6 (+ anti-Ifnar1 Ab)	Dakar 41525	Subcutaneous, intraperitoneal	Lesions in CNS, high viremia, decreased weight	[79]
Human STAT2 KI	ZIKV-Dak-MA	Subcutaneous	Viral RNA in brain	[16]
A129	MP1751	Subcutaneous	Lesions in brain, viral RNA in the brain	[80]
AG129	H/PF/2013	Footpad, intraperitoneal	Virus in brain, neurodegeneration, myofiber necrosis, inflammatory cell infiltration, pathology in brain	[18]
AG129	P6–740	Subcutaneous	Viral load in the brain, limb weakness, paralysis, increased neurological signs including locomotor hyperactivity, tremor, and seizure	[19]
AG129	MR 766	Intraperitoneal	Paralysis, neurodegeneration, acute encephalitis, viral antigen in brain and spinal cord	[81]
Ifnar^−/−^, Irf3^−/−^Irf5^−/−^Ifr7^−/−^ TKO	H/PF/2013, MR766	Subcutaneous, intravenous, intraperitoneal	Viral RNA in brain and spinal cord, increased neurological disease signs including hindlimb weakness and paralysis	[45]
**Eye**				
C57BL/6	PRVABC59	Subcutaneous	Chorioretinal lesions, induced local inflammation and cellular infiltration	[84]
C57BL/6, ISG15^−/−^	PRVABC59	Intravitreal	Chorioretinal atrophy with RPE mottling, increased multiple inflammatory and antiviral response genes in the retina, severe chorioretinitis coinciding with retinal cell death and higher virus replication in ISG15^−/−^ mice	[85]
C57BL/6, Ifnar1^−/−^	PRVABC59	Anterior segment of eyes, intraperitoneal	Increased intraocular pressure, chorioretinal atrophy, increased expression of inflammatory mediators, retinal ganglion cell death	[86]
**Lymph node**				
Rhesus macaque	PRVABC59, Brazil/ZKV/2015	Subcutaneous	Virus in lymph nodes in both paracortical regions, germinal centers, and cerebrospinal fluid, upregulation of proinflammatory and anti-apoptotic signaling pathways	[88]
Rhesus monkeys	ZIKV-BR	Intravenous	Activation of lymphoid tissues	[90]
Rhesus monkeys	PRVABC59	Subcutaneous	Virus in multiple lymphoid tissues, viral RNA in lymphoid and joint/muscle tissues	[89]
Pigtail macaque	Brazil_2015_MG	Subcutaneous	Increase of innate immune cells in blood, lymph nodes, and mucosal tissues, activation of monocytes in peripheral lymph nodes	[91]
Olive baboon	H/PF/2013, PRVABC59	Vaginal deposition of ZIKV-infected semen	Viral RNA in lymph nodes	[92]

## 5. Discussion

The selection of appropriate animal models for preclinical studies is essential for the successful development of antiviral vaccines or therapeutics. Particularly, mice and nonhuman primates have been commonly used in animal studies of ZIKV infection. However, each animal model displays slightly different clinical symptoms under ZIKV infection depending on the genetic background and/or the route of viral inoculation. Since immunocompetent wild-type mice are resistant to ZIKV infection under natural conditions, alternative methods such as high-dose injection of ZIKV or treatment with antibodies targeting IFN receptors have been used [79]. Alternatively, immunocompromised mice that are genetically modified to disrupt IFN signaling pathways have been used to study ZIKV infection [50,51]. Several studies have demonstrated that IFN-deficient immunocompromised mice are more susceptible to ZIKV infection and show more severe pathological symptoms than immunocompetent mice. Nonhuman primates have also been used; despite the high cost, group size limitations, and ethical concerns, they have gestational and fetal development processes more analogous to those of humans [31]. In nonhuman primates, subcutaneous or intravenous ZIKV administration induced a relatively similar pattern of lesions, while mice exhibited more severe pathologic symptoms following intravenous or intraperitoneal injection than with subcutaneous injection.

**Figure 2:**
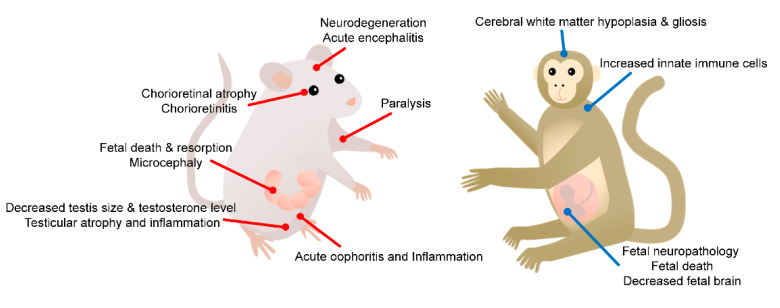
Tissue-specific pathologies of ZIKV infection in animal models.

Although different types of animal models show slightly different levels of clinical manifestation, a common finding is that ZIKV can infect multiple tissue types and induce tissue-specific symptoms (Figure 2). Indeed, ZIKV exhibits broad tissue tropism, including to maternal placenta, fetal brain, reproductive systems, adult brain, eyes, and lymph nodes, under diverse routes of infection. Vertical transmission of ZIKV from maternal placenta to fetal brain causes microcephaly or fetal death. Sexual transmission of ZIKV results in the infection of both male and female reproductive systems. Several studies have shown that ZIKV can persist in male semen, whereas female genital infection is more transient [68]. The central nervous system is also susceptible to ZIKV infection. Notably, ZIKV can replicate in the adult brain and spinal cord, causing neurological disorders such as paralysis or Guillain-Barré syndrome [8]. As part of the central nervous system, the optic nerve can disseminate ZIKV into the eye, leading to conjunctivitis or even glaucoma [86]. ZIKV can also spread to lymph nodes, causing lymphocyte accumulation, inflammation, and the activation of macrophage and B-cell subsets [89].

Since animal studies have shown that the extent of ZIKV pathology is dependent on the tissue tropism and route of administration, it is essential that we understand the tissue-specific symptoms and most relevant routes of ZIKV inoculation. Only then will researchers be able to accurately assess the efficacy and safety of vaccine candidates. If an animal model showing ZIKV infection only in the mother’s placenta or fetus is selected for a preclinical study, the effect of the vaccine candidate on other tissues will be veiled. This can lead to misunderstandings of the efficacy and safety of vaccine candidates and lead to the failure of clinical studies. Accordingly, it would be more appropriate to use immunodeficient mice, such as A129 or AG129 mice, for preclinical studies. Systemic inoculation of ZIKV, such as via intraperitoneal or intravenous routes, can induce broad disease manifestations, and subcutaneous inoculation can also cause systemic disease [18,19,23,50,51,72,80,81,93,94,95]. When immunocompetent mice are used for preclinical studies, the direct injection of ZIKV into the target organ, such as vaginal or intracerebral inoculation, may induce more pronounced symptoms for evaluating the vaccine efficacy [19,60,96]. Human STAT2 KI mice can be used as an alternative to immunocompetent wild-type mice [16]. Even subcutaneous inoculation of ZIKV into pregnant STAT2 KI mice resulted in higher levels of ZIKV RNA in the placenta and fetal head compared with wild-type mice. Although human STAT2 knock-in mice can recapitulate the hallmarks of human infection, this model needs further improvement as it requires a mouse-adapted ZIKV strain and exhibits low permissiveness to infection in adult mice. In addition, non-human primate models will be more useful for testing vaccine efficacy against certain diseases, such as Guillain-Barré syndrome, that are difficult to study in mouse models due to structural differences. This review comprehensively discussed the clinical symptoms of different animal models depending on the route of ZIKV inoculation and tissue-specific lesions. It will provide insight into selecting a suitable animal model and may suggest strategies for preclinical studies on evaluating ZIKV vaccines and therapeutics.

## Figures and Tables

**Table 1:** ZIKV vaccine candidates in clinical phase 1 and 2.

Vaccine Platform	Name	Immunogen	Phase	Sponsor
DNA	VRC5283	prM/E	2	National Institute of Allergy and Infectious Diseases (NIAID)
	GLS-5700	prM/E	1	GeneOne Life Science/Inovio Pharmaceuticals
	VRC-ZKADNA085-00-VP	prM/E	1	NIAID
mRNA	mRNA-1325	prM/E	1	Moderna
Inactivated virion	ZPIV	Whole virus	1	NIAID
	PIZV or TAK-426	Whole virus	1	Takeda
	VLA1601	Whole virus	1	Valneva Austria GmbH
Peptide	MV-Zika	prM/E	1	Themis Bioscience

## Data Availability

Not applicable.
